# Exploring the Characteristics and Behaviors of Nurses Who Have Attained Microcelebrity Status on Instagram: Content Analysis

**DOI:** 10.2196/16540

**Published:** 2020-05-26

**Authors:** Hanna Kerr, Richard Booth, Kimberley Jackson

**Affiliations:** 1 Western University London, ON Canada

**Keywords:** nursing, social media, professionalism, microcelebrity, Instagram, policy, influencer

## Abstract

**Background:**

Instagram is a social media platform that enables users to share images and videos worldwide. Some nurses have used Instagram to document their experiences as a nurse and have subsequently gained microcelebrity status—that is, a user who purposefully seeks to amass a substantive Web-based following and has become recognized as a niche area of interest.

**Objective:**

This study aimed to identify the characteristics and behaviors of microcelebrity nurses who act as influencers on Instagram and use their nursing profile to gain attention and presence on the Web.

**Methods:**

A qualitative, exploratory, nonparticipatory content analysis of media and text generated by a purposeful sample of 10 registered nurses who use Instagram and sustain a definable microcelebrity status was conducted. In this study, manifest and latent data were examined to gain an understanding of the characteristics and behaviors of nurses who have attained microcelebrity status on Instagram.

**Results:**

Data analysis revealed 5 themes of Instagram posts: (1) engaging Instagram users, (2) educational opportunities and insights, (3) nursing-related humor, (4) emotions experienced by nurses, and (5) media and narratives including patient details or work context. Messages were primarily positive in nature; however, multiple potential privacy, ethical, and professional issues were noted throughout the posted content.

**Conclusions:**

The findings of this study help to expand the current knowledge related to the use of social media platforms such as Instagram, especially in regard to the emergence of nurses who use this form of technology to achieve or maintain a microcelebrity status. This study calls for additional research on nurses’ attainment of microcelebrity status on social media as well as further policy development to adequately prepare nurses to navigate social media.

## Introduction

### Significance

Every month, more than one billion people use the photo- and video-sharing social media platform, Instagram [1]. As a mobile photo-sharing platform, Instagram users can post photos and videos that are shared with other Instagram users, customized through the use of image processes (ie, filters), textual captioning up to 2200 characters in length, and hashtags [2]. Instagram users interact with others through a dynamic process of *liking* and *commenting* on the posted media as well as *following* fellow users. The number of followers an Instagram user has is publicly displayed on a user’ s account page, regardless of the user’s privacy settings [3].

Owing to the interactive nature of Instagram and the ability to identify the number of followers a specific user possesses, a behavior demonstrated by some Instagram users of purposefully cultivating and amassing a substantial Web-based following has become a recognized phenomenon, that is, if a social media user gains both substantial attention and a distinct following within their group of followers, they are said to have attained *influencer* or *microcelebrity* status on social media [4-8]. Many individuals, including some nurses, have begun to purposefully leverage the connective powers of Instagram to generate microcelebrity persona through their Web-based presence.

At present, there are numerous Instagram accounts that exclusively feature content related to the nursing profession that has amassed sizable followings of more than 10,000 users, arguably pushing them toward microcelebrity status on Instagram [5,6,8]. However, given the potentially sensitive nature of the nursing role and related patient care, the use of Instagram by some users to post content related to the nursing profession is an area in need of deeper exploration. For instance, sharing of elements related to client care details and personal health information with the public raises potential concerns about the professional, ethical, and legal ramifications of such accounts and their related users.

Previous research examining the motivations of Instagram users and their use of social media platforms has found aspects related to (1) attention seeking; (2) social support; (3) belonging; (4) social interaction; (5) documentation; (6) learning about other users’ lives; and (7) self-expression, as being important to users [9-13]. Within the nursing literature, several reasons have been explored regarding why nurses use social media platforms such as Instagram. The use of social media by nurses may assist in providing benefits for both nurses and patients, in part because of the ability to rapidly share information, assist users who are geographically isolated, and engage in health care planning [14-24].

Although social media have several potential benefits for nurses, challenges have also been identified with social media use in the profession. Social media posts can be disseminated to vast audiences immediately, which can have unintended results, especially if posts lack sensitivity or breach personal health information [16,17,20,22,23,25-27]. Given the subtle and encompassing nature of social media platforms to provide users with a false sense of security and privacy, the risk for health care providers to unintentionally cross professional boundaries on the Web is a contemporary reality for all nurses [17,18,23,25,28,29].

The consequences for nurses who breach patient privacy or confidentiality on social media are significant, including possible job termination, investigation and reprimand from nursing board(s), monetary fines, and loss of licensure. Furthermore, nurses may face civil or criminal charges, resulting in a potential jail time [17-20,28]. In recent years, there have been several instances of nurses violating the Health Insurance Portability and Accountability Act (HIPAA) policies on social media and being disciplined for their actions [20,26,27]. In Texas, a pediatric nurse posted comments on Facebook about a patient with measles who she cared for. No advertent personal information was disclosed; however, the nurse was terminated for HIPAA violation [26].

As a result of ongoing concerns related to unprofessional social media use, nurses are advised to use social media with caution and awareness regarding what they are posting [17,19,20,28]. Nurses are prohibited from posting any images or videos depicting patients or any content that would enable a patient to be recognized. In addition, nurses are warned that it is possible to violate privacy policies without explicitly disclosing patient information [17,19,20,28]. In fact, most instances of nurses violating patient privacy and confidentiality on social media are accidental in nature [17,28]. Finally, nurses are advised to refrain from interaction with patients over social media or use substantial caution in doing so [17,18,28,30-32].

Although the current body of literature examining social media usage by nurses contains insights into both positives and negatives of social media usage, there is an absence of research exploring the growing presence of microcelebrities in the profession who use platforms such as Instagram to showcase their interpretation of the profession. To proactively ensure the safety and privacy of patients and health care professionals in the coming years, it is necessary to examine the current characteristics and behaviors of nurse influencers who use Instagram to expose various elements of the nursing profession, especially those with significant Web-based followings.

### Purpose of the Study

The purpose of this study was to identify the characteristics and behaviors of nurses who have attained microcelebrity status on Instagram and use their nursing profile to gain attention and presence on the Web.

## Methods

### Overview

This study was an exploratory, nonparticipatory content analysis of publicly available data found on Instagram, guided by Graneheim and Lundman’s qualitative content analysis framework [33]. The framework developed by Graneheim and Lundman was selected for the study to enable researchers to gain a rich understanding of information by analyzing both manifest and latent data [33]. In this study, data were examined to identify the characteristics and behaviors of *microcelebrity Instagram users* [33]. The underlying meaning of posts was examined to gain an understanding of the types of messages being projected by microcelebrity Instagram users.

An initial step in Graneheim and Lundman’s content analysis framework is the identification of the unit of analysis [33]. In this study, the unit of analysis was a textual description of the microcelebrity Instagram users’ Instagram posts, including the direct caption and the researcher’s description of each post. Once created, the unit of analysis was divided to form meaning units. The meaning units for this study were written descriptions of each individual Instagram post and the exact accompanying caption. The meaning units were condensed to summarize the manifest content of each Instagram post. After the manifest content emerged, abstraction took place, in which the summarized meaning units were further analyzed for their latent content and labeled with a code. Codes were grouped into subcategories and categories [33]. The categories were reviewed and revised, as emergent themes were identified [33].

### Ethics Approval

The Western University Health Sciences Research Ethics Board deemed this study did not require ethical clearance for completion.

### Sample

Purposive sampling was used to enable variation in identifying the characteristics and behaviors of nurses with active microcelebrity status on Instagram. To be included in the study, the following needed to be met: (1) the user’s Instagram account had to be publicly available, possessing a minimum of 10,000 followers; (2) the 10 most recent posts from the user must have been created between 2017 and 2018; (3) all content should be written in English; (4) the majority of the user’s content had to be focused on the nursing profession; and (5) the user must have self-identified as a registered nurse in their Instagram account description. Owing to the exploratory nature of this study, the sample was limited to registered nurses. A small sample enabled researchers to gain a rich understanding of the phenomenon within this group.

A priori, it was estimated that analysis of 10 to 15 microcelebrity Instagram accounts would result in data saturation. The amount of data to be collected from 10 to 15 microcelebrities (with 10 posts per individual) was deemed to be sufficient and comparable with other studies that used purposeful samples of social media data [34-36].

To obtain the sample, hashtag and keyword searches were performed on Instagram using the following search terms: *#nurse(s), #registerednurse, #rn* as well as *nurse(s), registerednurse,* and *rn.* These terms were selected to aid the identification of relevant Instagram accounts. When a hashtag is searched on Instagram, results consist of publicly available Instagram posts that have been tagged with the hashtag. Posts are displayed as *top posts* and *most recent* posts. *Top posts* populate hashtags that are trending, displaying 9 of the most liked posts containing that hashtag [3]. For each search, the 9 *top posts* for every hashtag were selected, and the Instagram user who created the post was identified and examined for study eligibility. Sampling was limited to the *top posts* search results, as the study aimed to identify Instagram users with active microcelebrity status.

Following the hashtag search process, an Instagram keyword search was conducted to identify additional eligible Instagram users. When an Instagram keyword search is conducted, the results populate, as a list of Instagram accounts that are relevant to the keyword searched. The first 10 results of each keyword search were assessed for study eligibility. Through the sampling process, 10 Instagram accounts were selected for the study. To protect the privacy of Instagram users, each account was randomly assigned a participant code number between 01 and 10.

### Data Collection

The data collection and analysis framework for the study was developed from Graneheim and Lundman’s [33] framework for qualitative content analysis. The data for this study consisted of each of the 10 microcelebrity Instagram user’s 10 most recent posts (images, videos, and captions). To collect data, the researcher examined each Instagram user’s posts individually. All study data were collected between September 2017 and August 2018. Each microcelebrity Instagram user was identified, and their 10 most recent posts were labeled in reverse chronological order. For each Instagram post, the researcher copied and pasted the entire caption to a secured word document. Then, the researcher described the manifest content of the post’s photo or video in the written text. For example, the manifest content of microcelebrity Instagram user 10’s, post 7 was captured as follows:

A collage of three images of the microcelebrity Instagram user. Image one: Microcelebrity Instagram user walking towards a retail store with a shopping cart. Image two: Microcelebrity Instagram user sleeping in a bed. Image three: Microcelebrity Instagram user sitting on a couch holding a glass of wine, an ice cream carton, and a pizza box. The heading of the collage states, “What nurses do on their days off...” The caption states, “This is my life! ... Please tell me I’m not alone.”

Microcelebrity Instagram user 10, Post 7

The data collection process was completed for the 10 most recent posts of each of the 10 Instagram users, totaling 100 Instagram posts for analysis.

### Data Analysis

From the unit of analysis, meaning units were created by dividing the text into each microcelebrity Instagram user’s content and further subdividing them into individual Instagram posts. Thus, the meaning units for this study comprised a written description of each Instagram post. The meaning units were then condensed to summarize the manifest content of each Instagram post [33]. Through this process, the researcher abridged the direct meaning of both the photo or video and caption into a shortened textual description.

Following summarization, meaning units were exported to NVivo 12 (QSR International) for further analysis. Then, the process of abstraction took place, in which the summarized meaning units were analyzed for their latent meaning and labeled with a code. Once each post was coded, the codes were analyzed for similarities and differences and grouped into subcategories and then into categories. The categories were then reviewed, discussed, and revised. Once the revision process was complete, emergent themes were identified [33].

## Results

### Overview

Through data analysis, 5 themes emerged: (1) *engaging Instagram users,* (2) *educational opportunities and insights,* (3) *nursing-related humor,* (4) *emotions experienced by nurses,* and (5) *media and narratives including patient details or work context.* Seven of the microcelebrity Instagram users appeared to be female, and 3 microcelebrity Instagram users appeared to be male. Three of the microcelebrity Instagram users stated that they live in the United States of America, whereas the geographic locations of the remaining microcelebrity Instagram users were unknown.

### Engaging Instagram Users

Commonly, microcelebrity Instagram users interacted with followers by generating discussion within their posts. Multiple microcelebrity Instagram users posted questions related to the nursing profession or clinical knowledge and encouraged responses in the post’s comment field. For example, microcelebrity Instagram users encouraged discussion related to dealing with a negative workplace culture and strategies to find a new nursing job. In addition, microcelebrity Instagram user 05 created multiple problem-solving posts that encouraged users to test nursing-related knowledge (Microcelebrity Instagram user 05, posts 2 and 10).

Along with a generic discussion of the profession or clinical knowledge, most microcelebrity Instagram users also created personalized brands based on some elements of their unique physical, cultural, or personality characteristics (eg, gender, ethnicity, and religion) and reinforced elements of this personalized brand within their Instagram posts. For example, microcelebrity Instagram user 04 repeatedly engaged in self-branding by highlighting the personal characteristics that made him unique. Microcelebrity Instagram user04 stated:

When people say, “What’s a nurse?” They think of Florence Nightingale, a female wearing a little hat, white dress with a stethoscope. They don’t automatically think of someone who’s a Bilingual/Hispanic Male or a different culture ethnicity all Tatted covered with Ink...thats a MAN of GOD.

Post 7

Multiple microcelebrity Instagram users engaged in other branding activities by encouraging users to purchase individualized products that they developed or endorsed. Furthermore, promoting external merchandise and products was common, as 8 of the microcelebrity Instagram users promoted at least one merchandise or product that was not their own creation. For instance, microcelebrity Instagram users promoted businesses selling nursing-related uniforms, equipment, or services as well as non-nursing-related companies (eg, Cherokee Uniforms, Peloton, and Johnson & Johnson). In all these examples, it was unclear if the microcelebrity Instagram users were compensated for creating posts.

### Educational Opportunities and Insights

Nursing-related educational opportunities and insights were a prominent theme of Instagram posts. Microcelebrity Instagram users encouraged followers to attend nursing-related educational events and posted photos of themselves at nursing conferences. In addition to promoting formal educational opportunities, multiple microcelebrity Instagram users shared their insights related to clinical nursing skills. For example, microcelebrity Instagram user 05 posted medication dosage questions, along with information about the medication, and encouraged users to complete the problem and comment on the solution. In addition, microcelebrity Instagram users provided their opinions on the various roles that nurses undertake. For example, microcelebrity Instagram user 04 posted 3 video clips in which operating room nurses discuss their responsibilities. In the videos, operating rooms were shown to appear to be in use, including patients on operating tables. It is unclear if the videos are real or performed; however, the videos appear realistic. Finally, only 2 posts related to obtaining formal nursing education at an undergraduate or master’s level, both of which shared a general motivational message about persevering through the challenges associated with nursing school.

### Nursing-Related Humor

In this study, the use of humor was evident in several Instagram posts. Frequently, humor was used in a manner that may only be understood by those with the contextual knowledge that comes with working as a health care professional, such as:

Remember don’t over resuscitate your self with turkey and beer or you might end up with Abdominal Compartment Syndrome!

Microcelebrity Instagram user 03, Post 3

Another example was related to the experiences that nurses may encounter at the workplace. Microcelebrity Instagram user 01 posted an image of a white background with overlaying text:

“What were his respirations?” First of all, I haven’t counted respirations since nursing school.

Post 8

The caption reads:

Too slow, too fast, normal, prepare to intubate. Those are the rates.

Microcelebrity Instagram user 01, Post 8

To fully understand these forms of humor, readers must possess the requisite knowledge of medical terminology as well as the contextual knowledge associated with working as a health care provider.

Humor was observed in multiple Instagram posts, as microcelebrity Instagram users described potentially stressful situations. Microcelebrity Instagram user 08 posted a series of memes, depicting a professional basketball player holding his arms out, mouth open, appearing shocked, and disgruntled during a game. The headings were as follows:

When ICU refuses to take report because the patient is “too unstable.”

Microcelebrity Instagram user 08, Post 5

When your A&Ox4 patient decides they’re too tired so they just pee in the stretcher.

Microcelebrity Instagram user 08, Post 5

The caption further read:

I wish I could say all of the above scenarios are made up-but no no, they’re all real life.

Microcelebrity Instagram user 08, Post 5

Similar to the previous examples, users require contextual and clinically based knowledge to interpret the meaning of this post.

### Emotions Experienced by Nurses

Inspiration, motivation, and encouragement were the most prominent emotions displayed by Instagram users, as every microcelebrity Instagram user shared at least one post demonstrating these emotions. Several posts were positive in nature, as microcelebrity Instagram users presented optimistic captions or portrayed nurses as heroic figures. For example, microcelebrity Instagram user 02 expressed gratitude by stating:

There are many forms of family...For me, my work family is as much a part of my life as my real one...There’s something about the few quiet moments on a morning like this before the chaos starts when you’re reminded to give thanks for countless reasons.

Post 9

In addition to the positive emotions associated with the nursing profession, microcelebrity Instagram users described the challenges they experienced while nursing. The challenges described by microcelebrity Instagram users included mental and physical exhaustion, learning how to prioritize tasks, manage time, deal with workplace bullying, and shiftwork. Although many challenges associated with nursing were identified, every Instagram post that discussed a challenge concluded with a positive message. For example, microcelebrity Instagram user 02 stated:

Today was, legitimately, a “Top 3 Worst Shifts I’ve Ever Had as an ICU Nurse” kind of day. Nothing went right...& things just got worse by the hour...I’m exhausted...But no matter how bad the day gets—& trust me, in our world, it can get ugly—only your work family knows how to make you crack a smile...I genuinely believe that the long, ugly days & crazy hours impact how you see the world. Smile because you have love. Smile because you have life. Smile because life is precious, & every moment with loved ones matters...Give thanks, even after a long & torrid day.

Post 8

In these posts, microcelebrity Instagram users consistently shared messages of encouragement, despite experiencing challenging situations associated with the nursing profession.

### Media and Narratives Including Patient Details or Work Context

Multiple Instagram posts depicted scenarios in which the photo or video appeared to have taken place in a workplace setting or potentially include patient information. For instance, multiple Instagram posts appear to depict patients in the hospital, although the sources of the images are not cited. microcelebrity Instagram user 03 posted an image of a patient’s abdomen undergoing a surgical procedure, providing education on abdominal compartment syndrome in the caption. It is unclear if the patient consented to have this photo taken or if consent was obtained to post the image on Instagram. In another post, microcelebrity Instagram user 03 posted an image of a patient sitting in a stretcher, covered in dried blood, smiling, and surrounded by hospital staff members. The caption was unrelated to the image, asking volunteers for a trauma course. In addition, microcelebrity Instagram user 04 posted a series of video clips showing what appeared to be multiple patients on operating tables while receiving preprocedure treatments. No qualification was made in the post as to whether the patients depicted in the image were actually real or standardized patient actors.

Furthermore, several of the microcelebrity Instagram users’ Instagram posts consisted of images that appear to have been taken in workplaces, with no patients seen. Multiple posts depicted microcelebrity Instagram users dressed in work attire while in hospital settings; other posts showed patient care areas with no patients present.

## Discussion

### Principal Findings

The findings of this study have identified a gap between nursing research, policy, and practice. Previous research has recommended the integration of social media with the nursing profession; however, policies continue to advise nurses to keep their professional lives separate from any social media use [14,28,37-40]. Further inquiry should be conducted to determine why this gap exists and identify how elements of policy and practice can be integrated in a safe and efficient manner.

All 10 microcelebrity Instagram users attempted to engage with followers, which supports previous research suggesting that a primary motive for Instagram use is social interaction [9]. Eight microcelebrity Instagram users directly participated in self-branding or merchandise solicitation. In particular, microcelebrity Instagram user 04 consistently promoted their personal brand, attempting to generate a distinct persona through reinforcement of this brand to their followers. It appears that microcelebrity Instagram user 04 may have been attempting to use his charismatic nature to help foster a sizable Web-based following by capitalizing on their personal brand as a *unique selling point* [6,7,41]. Furthermore, microcelebrity Instagram users consistently engaged with followers by generating discussion, which may have been in an attempt to gain social support and encourage feelings of belonging, as this has been found to be a primary motivation for Instagram use [9-11].

An interesting finding of this study was that many microcelebrity Instagram users used Instagram to share learning opportunities and describe their understanding of nursing-related clinical information. For example, microcelebrity Instagram users challenged followers to answer questions about medication administration. In addition, microcelebrity Instagram users described their perspective on the roles and responsibilities that nurses have in specific work environments, including areas such as the operating room. Sharing insights about nursing-related learning has not previously been identified as a primary reason for Instagram use. The findings from this study suggest that microcelebrity Instagram users may also use Instagram to promote educational opportunities such as courses and conferences. The reasons why microcelebrity Instagram users promoted these learning opportunities are unknown.

Humor was frequently demonstrated by microcelebrity Instagram users, often used in a manner that required the reader to have contextual knowledge to understand clinical abbreviations and terms used in the caption, in conjunction with the corresponding image. In addition, self-enhancing humor appeared to be used as a way to manage potentially stressful situations [42]. For example, microcelebrity Instagram user 08 posted a series of memes that described situations that could be experienced in nursing. However, depending on the reader’s contextual understanding, the post may be interpreted as a form of mockery for not following the directions or advice given by the health care provider.

It was also common for microcelebrity Instagram users to express their emotions through Instagram posts, which has been previously identified as a motive for Instagram use [9,11]. However, previous literature suggests that self-expression on Instagram is achieved primarily through the process of creating, editing, and posting images [9,11]. In this study, self-expression was often conveyed through heartfelt captions rather than the images themselves. Images included in the posts with meaningful captions frequently appeared to be unrelated to the caption. Most often, the unrelated images were of the microcelebrity Instagram user standing alone and smiling. In these posts, it appears as though microcelebrity Instagram users were primarily using the captions to express themselves, as opposed to creating unique images as a form of self-expression.

Five posts generated by microcelebrity Instagram users appeared to potentially depict patients, although the sources of the images and videos are unknown. As such, it is unclear if the images and videos contained patients or actors. The overwhelming consensus from nursing practice regulators is that nurses should refrain from posting any content on social media that contains patients or patient information [28,37-40]. Similarly, previous research advises nurses to refrain from posting patient information on social media [14,16-18,20,28,37-40]. However, an image posted by microcelebrity Instagram user 03 showed an open abdomen during a surgical procedure. Similarly, microcelebrity Instagram user 04 posted a series of videos showcasing an operating room environment, with what appeared to be patients awaiting surgical procedures. The uncertainty as to whether these posts show real patients or standardized patient actors demonstrates the need for further research to guide education and policy development. If the posts are depicting actual patients, there needs to be further research regarding the response of other clinicians witnessing this behavior as well as regarding how infection control practices may be compromised by staff taking pictures amid surgical procedures.

Finally, several posts showed the microcelebrity Instagram user posing in a workplace setting, dressed in work attire, with no overt patient health information displayed. At present, professional nursing guidelines do not specify whether or not it is appropriate to post an image of oneself in a workplace [37-40]. However, the Registered Nurses Association of Ontario (a professional association for Ontario nurses, who often publish clinical practice guidelines) [40] suggests that if there is uncertainty if a post is appropriate, the user should refrain from posting. Given that multiple microcelebrity Instagram users post workplace-based images, further work should be completed to determine how health care organizations interpret the depiction of their facilities via social media channels, especially when these images are instantaneously amplified by the significant following possessed by microcelebrity Instagram users.

### Nurses’ Social Media Use

Over the last decade, practical recommendations and policies developed by both researchers and regulatory bodies have begun to emerge in an effort to help mitigate social media–related risks by expressing the expectations of nurses and reinforcing best practice guidelines [14,17,19,28,37-40]. Much of the available advice regarding nurses’ social media use portrays social media use in a negative manner, focusing on what not to do [21]. However, some literature does exist on how nurses can use social media in a positive manner. For example, nurses are encouraged to build a personal brand and express their opinions in health-related conversations on social media platforms, provided they are aware of and comply with privacy as well as relevant workplace policies [19,43]. In this study, multiple microcelebrity Instagram users developed personal brands and voiced their opinions on various nursing-related topics via Instagram. The findings of this study also demonstrate that nurses may be violating regulatory policies, as evidenced by images and videos of what appear to be patients. The findings of this study demonstrate a need for further guideline development that focuses on what nurses should be doing on social media to maintain professionalism and protect patients’ rights.

### Implications for Nursing Education and Practice Policies

The findings of this study highlight the need for further education and practice policy development with regard to nurses’ Instagram use. Incongruencies currently exist between (1) how recommendations for integrating social media into nursing practice are enacted by some nurses; (2) the current guidelines for social media use by nurses; and (3) the ways in which nurses use social media, especially those possessing Web-based microcelebrity status. Although previous research has recommended that social media be integrated with nursing practice, current policies strongly urge nurses to avoid any and all contact with patients over social media [37-40]. In contrast, the findings of this study demonstrated that nurse microcelebrities actively engage and interact with thousands of Instagram users and generate personalized interpretations of the nursing profession via social media. In addition, in this study, multiple microcelebrity Instagram users posted images or videos that appeared to have depicted patients receiving care. Although the sources of the posts are not specified, the content of these posts directly contrasts existing guidelines for nurses’ social media use. For the nursing profession, it is imperative for nursing education, professional regulatory bodies, and employers to develop more robust and dynamic policies and guidelines related to the appropriate use of social media within the profession [16,21], especially with the growing presence of Web-based nurse microcelebrities.

At present, a gap exists in the policy regarding Instagram and other social media use by nurses who have attained a microcelebrity status. The findings of this study suggest that nurses who have achieved microcelebrity status may use Instagram for a variety of purposes, and there needs to be clarity in what is considered appropriate professional behavior. Given the rapid evolution of social media, it is imperative that both education and policy initiatives make efforts to maintain relevancy as related to new usages of these kinds of technology, including nurses who actively use these platforms to seek Web-based microcelebrity status.

### Limitations

A limitation of the study was the noninteractive nature of the research, whereby only secondary analysis of preexisting Instagram photos and textual content were analyzed. Owing to the methodology and ethical requirements of the study, no participants examined in this study were contacted to comment on the findings emerging from the data analysis. Thus, ensuring appropriate representation of data, as opposed to the researcher’s interpretation of data, was a challenge of this qualitative content analysis [33].

Finally, this study focused exclusively on registered nurses who had achieved a level of microcelebrity status only on Instagram. Although Instagram is one of the most popular social media platforms in 2019, different or more nuanced findings may have emerged if nursing microcelebrities who used other social media platforms (ie, YouTube and Twitter) were included in the study [1].

### Future Directions

The findings of this study support the need for further research in several domains related to nurses who have attained microcelebrity status on Instagram. Further research should be completed in both the qualitative and quantitative paradigms to develop a well-rounded understanding of the concept of nurses seeking microcelebrity status on social media. Future explorations should also be conducted to determine who are following nurse microcelebrity Instagram user accounts and their reasons for doing so. The examination of merchandise endorsement as well as the reactions of microcelebrity Instagram users’ followers to Instagram advertisements is also a worthwhile area for subsequent exploration. Similar research may be conducted on alternate groups of health care professionals, such as physicians, to identify if similar behaviors occur outside of the nursing profession. Finally, there is an urgent need for further research on the risks associated with nurses posting images and videos depicting patient care on Instagram.

### Conclusions

The findings of this study suggest that posting characteristics and behaviors of registered nurses who have attained microcelebrity status on Instagram include (1) *engaging Instagram users,* (2) *educational opportunities and insights,* (3) *nursing-related humor,* (4) *emotions experienced by nurses,* and (5) *media and narratives including patient details or work context.*

Implications exist for nursing professionals in terms of potential privacy, professionalism, and ethical challenges associated with social media use. As social media platforms continue to thrive and evolve, nurses must be able to effectively use social media while maintaining professionalism. Nursing practice policies and guidelines must be updated to include recommendations pertinent to nurses with microcelebrity status, to maintain nurses’ professionalism and to protect patients’ safety.

## Abbreviations

HIPAA: Health Insurance Portability and Accountability Act
